# Molecular Analysis of HLA Genes in Romanian Patients with Chronic Hepatitis B Virus Infection

**DOI:** 10.3390/cimb46020067

**Published:** 2024-01-24

**Authors:** Adriana Tălăngescu, Bogdan Calenic, Dan Florin Mihăilescu, Maria Tizu, Ion Marunțelu, Alexandra E. Constantinescu, Ileana Constantinescu

**Affiliations:** 1 Immunology and Transplant Immunology, Carol Davila University of Medicine and Pharmacy, 258 Fundeni Avenue, 022328 Bucharest, Romania; adriana.oprea@drd.umfcd.ro (A.T.); maria.tizu@drd.umfcd.ro (M.T.); ionutz.marion@gmail.com (I.M.); alexandra.constantinescu08@gmail.com (A.E.C.); ileana.constantinescu@imunogenetica.ro (I.C.); 2 Centre of Immunogenetics and Virology, Fundeni Clinical Institute, 258 Fundeni Avenue, 022328 Bucharest, Romania; 3 Department of Anatomy, Animal Physiology and Biophysics, Faculty of Biology, University of Bucharest, Splaiul Independenței Street, No. 91–95, 050095 Bucharest, Romania; d.f.mihailescu@gmail.com

**Keywords:** next-generation sequencing (NGS), human leukocyte antigens (HLAs), hepatitis B virus (HBV), amino acid residues

## Abstract

Hepatitis B, a persistent inflammatory liver condition, stands as a significant global health issue. In Romania, the prevalence of chronic hepatitis B virus (CHB) infection ranks among the highest in the European Union. The HLA genotype significantly impacts hepatitis B virus infection progression, indicating that certain HLA variants can affect the infection’s outcome. The primary goal of the present work is to identify HLA alleles and specific amino acid residues linked to hepatitis B within the Romanian population. The study enrolled 247 patients with chronic hepatitis B; HLA typing was performed using next-generation sequencing. This study’s main findings include the identification of certain HLA alleles, such as DQB1*06:03:01, DRB1*13:01:01, DQB1*06:02:01, DQA1*01:03:01, DRB5*01:01:01, and DRB1*15:01:01, which exhibit a significant protective effect against HBV. Additionally, the amino acid residue alanine at DQB1_38 is associated with a protective role, while valine presence may signal an increased risk of hepatitis B. The present findings are important in addressing the urgent need for improved methods of diagnosing and managing CHB, particularly when considering the disease’s presence in diverse population groups and geographical regions.

## 1. Introduction

Hepatitis B, a chronic inflammatory liver disease, is a major worldwide health concern, with 3 million new infections and over 1 million deaths each year (combined with hepatitis C). The infection can be both acute or chronic and spreads horizontally through contact with biological fluids such as saliva, blood, semen, tears, vaginal fluids, or perinatal fluids at childbirth, transferred from mother to newborn. The diseases can be easily prevented with a vaccine, usually given soon after birth; however, if left untreated, chronic hepatitis B can lead to life-threatening complications such as liver cancer or cirrhosis [1]. In Romania, the rate of chronic hepatitis B virus (CHB) infection is notably high, with 4.4% of the population testing positive for HBs antigen and 27.0% for anti-HBc antibodies. These figures are among the highest in the European Union, underscoring a critical need for the implementation of comprehensive strategies focused on the prevention, early detection, and effective treatment of HBV infection within the country [2].

To date, the mechanisms responsible for hepatitis B virus (HBV) pathogenesis remain unclear, with a complex interplay of viral, environmental, and host genetic factors determining the progression or the regression of the disease. In this context, correlations between HLA genes and disease severity, response to vaccination or treatment, and infection susceptibility have been widely studied across multiple populations and geographical areas. Human leukocyte antigens (HLAs), encoded by genes within the major histocompatibility complex (MHC), are integral transmembrane molecules that hold a central position in the intricate landscape of the human immune system. Among their major roles, HLA class I proteins are pivotal in binding endogenous peptides and subsequently orchestrating their presentation to CD8+ T cells, potentially culminating in the cytotoxic annihilation of the host cell upon activation of lymphocytes. Conversely, HLA class II proteins exhibit a more discerning expression pattern, being restricted to antigen-presenting cells (APCs) and B-lymphocytes, where their primary function revolves around the presentation of exogenous peptides, a process tailored for the activation of CD4+ T cells. These multifaceted roles position HLAs as fundamental mediators of immune recognition, immune regulation, and the crucial delineation between the self and non-self in immune responses.

The HLA genotype has been observed to play an important role in modulating the progression of hepatitis B virus infection, suggesting that specific variants of the HLA genetic profile can influence the outcome of the infection. Previous association studies indicate that specific alleles, such as DQA1*05:01, DQB1*03:01, and DRB1*11:02, are linked to an increased susceptibility to HBV infection or, later on, disease progression [3,4]. Conversely, alleles in both class I (A*03:01) and II (DRB*13:01, DRB1*13:02) are associated with a protective effect or enhanced viral clearance [5,6]. Numerous global studies underscore the substantial diversity in associations between HLA genotypes and hepatitis across different populations. For example, Chinese patients exhibit heightened hepatitis risk with HLA B*48 and DRB1*48 genotypes [7], whereas acute hepatitis B patients in the same population have lower occurrences of HLA-DRB1*11:01/11:04 and HLA-DQA1*03:01 compared to those with chronic hepatitis [8]. In Japanese patients, specific variants in HLA-DP and HLA-DQ genes are strongly linked to chronic HBV [9,10], while Turkish patients with chronic HBV notably exhibit elevated expression of HLA B*35 [11]. In Iran, the frequency of the B*52 allele is higher among chronic HBV patients than healthy carriers [12]. Among Caucasian patients, HLA B*08 and B*44 are associated with increased viral persistence [13], whereas HLA B*58, DRB1*13:01, and DRB1*13:02 genotypes provide protective effects for individuals with chronic HBV [5,14]. An extensive study conducted in Gambia, Africa, revealed that DRB1*13:02 is associated with protection against persistent HBV infection [15]. Very few studies have been done in Eastern Europe in general and in the Romanian population in particular regarding the association between HLA and hepatitis B. In Transylvania, Romania, a univariate analysis indicated a notable correlation between HBV infection risk and the presence of the HLA-DRB1*03 and HLA-DQB1*05 alleles. Conversely, the HLA-DRB1*01 allele was identified as providing a protective effect against HBV infection [16].

The primary aim of this study is to identify HLA alleles and HLA amino-acid residues associated with hepatitis B within the Romanian population. To achieve this goal, we will employ a significant study group and massively parallel sequencing technology in our analysis method.

## 2. Materials and Methods

### 2.1. Patients and Controls

**Figure 1:**
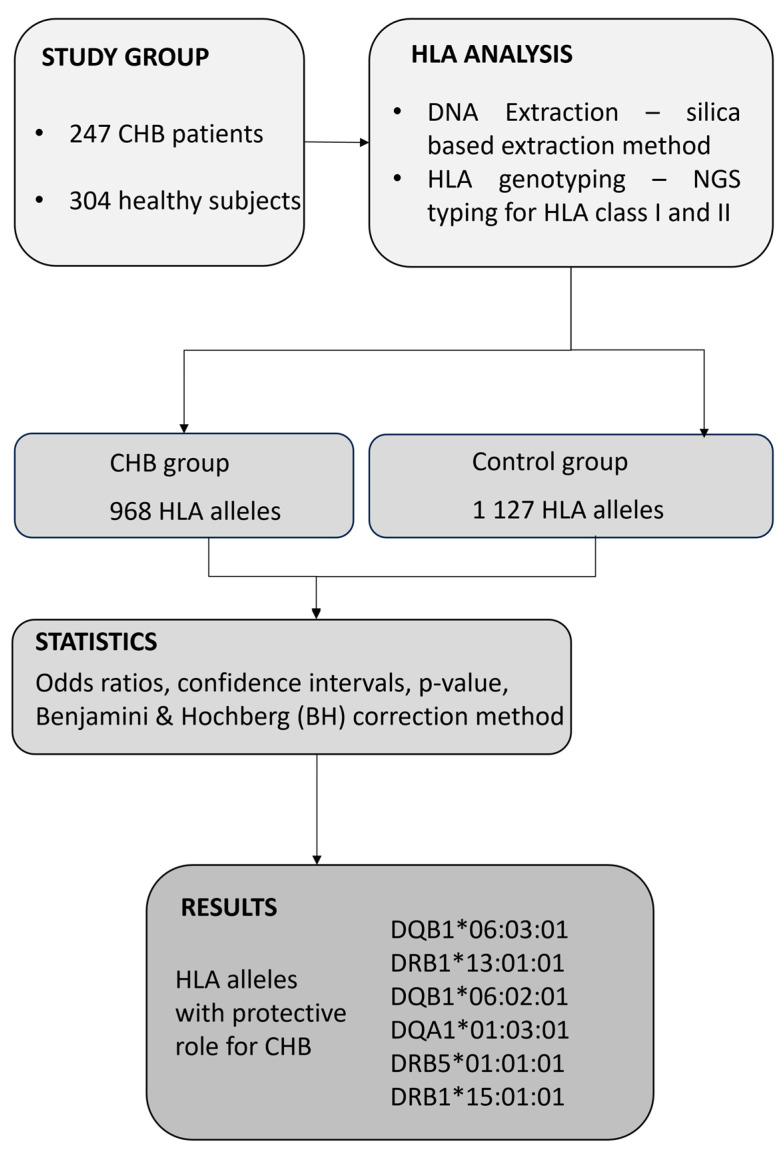
Flow chart of the present study design.

The present study was conducted in accordance with the Declaration of Helsinki (2013 version) and was approved by the Commission of Bioethics at the Fundeni Clinical Institute, Romania (No. 28640/25.05.2022). For collecting biological samples as well as for all medical procedures performed during the study, all participants signed an informed consent statement. In this study, we consecutively selected 247 patients with chronic HBV infection, defined as the presence of HBsAg and HBV DNA for more than six months, as identified at Fundeni Clinical Institute, Department of Gastroenterology and Hepatology, between September 2022 and October 2023. The study also included 304 healthy control subjects who were bone marrow volunteer donors. None of the donors reported a personal history of cancer or any HLA-associated disease, including chronic infections or autoimmune diseases. All of the study subjects were Caucasian, a single ethnic population. Patients were included in this study if their age was over 18 years. The diagnosis of chronic HBV infection was based on well-established biochemical and virological parameters. Thus, each patient was routinely analyzed for biochemical markers such as alanine aminotransferase (ALT), gamma glutamyl transpeptidase (GGT), aspartate aminotransferase (AST), alkaline phosphatase (ALP), total bilirubin (TB), and direct bilirubin (DB) using well-established enzymatic methods. Virological markers, HBs antigen (HBsAg), anti-HBs antibodies, HBe antigen (HBeAg), anti-HBe antibodies, anti-HBc antibodies (including total anti-HBc antibodies and anti-HBc IgM), and α-fetoprotein (AFP) were quantified through chemiluminescence. The following exclusion criteria were employed in the study: patients younger than 18 years, positive for co-infection with human immunodeficiency virus (HIV), hepatitis C virus (HCV), hepatitis D virus (HDV), hepatitis A virus (HAV), autoimmune hepatitis (AIH), and other liver diseases. All participants were Caucasian in the current study. A flow chart explaining the study design is presented in Figure 1. 

### 2.2. Sample Collection and DNA Extraction

To perform DNA extraction, we employed 200 µL of whole blood together with the QIAmp DNA Blood Mini^®^ kit (QIAGEN, Hilden, Germany), resulting in a 200 µL eluate. The DNA extraction process was based on a silica membrane method. Before extraction, each sample was vigorously vortexed, and we combined 200 µL of whole blood with protease and lysis buffer. Subsequently, these samples were incubated at 56 °C for 15 min to facilitate efficient lysis. Once the cell membranes were disrupted, releasing the DNA, we introduced alcohol to induce DNA precipitation. These prepared samples were then transferred to specialized tubes containing silica membranes. During centrifugation, the DNA adhered to the membrane due to differences in electrical charge. Following subsequent steps for DNA purification and washing, we detached the DNA from the silica membrane by adding an elution buffer to neutralize electrical charges. The resulting DNA was carefully collected into separate tubes and stored at −18 °C until required. The concentration and purity of the DNA were assessed using an IMPLEN nanophotometer. Solutions with an A260 nm/A280 nm ratio within the 1.7 to 1.9 range were deemed acceptable, signifying solution purity and ensuring a minimum DNA concentration of >20 ng/µL.

### 2.3. HLA Genotyping Using NGS

HLA genotyping for 11 loci, including HLA class I (HLA-A, HLA-B, HLA-C) and class II (HLA-DRB1, HLA-DRB3, HLA-DRB4, HLA-DRB5, HLA-DQA1, HLA-DQB1, HLA-DPA1, and HLA-DPB1), was conducted using next-generation sequencing reagents provided by Immucor (Mia Fora NGS Flex, Norcross, GA, USA), following the manufacturer’s instructions. For the long-range PCR amplification of HLA genes, genomic DNA with a concentration ranging between 5 and 15 ng/µL and an OD 260/280 ratio of 1.65–2.0 was utilized. After amplification, the PCR products were enzymatically cleaved into fragments, underwent end repair, and were tailed with an ‘A’ at the 3′-end to enable the ligation of index adaptors, which featured a 5′-T-overhang. Each sample was purified and then ligated with index adaptors containing unique barcode sequences, essential for the subsequent binding of sequencing primers and facilitating the attachment of the fragments to beads in later steps. These unique barcodes served the purpose of identifying different samples and/or loci during the final sequence analysis. The samples were further purified and size-selected using the Pippin Prep system (Sage Science, Inc., Beverly, MA, USA), which specifically chose DNA fragments falling within the desired size range of 500–900 base pairs. The library’s concentration was quantified using the Qubit™ dsDNA BR Assay kit and the Qubit Fluorometer (Thermo Fisher Scientific, Waltham, MA, USA). Prior to loading onto the NGS Illumina platform, the genomic library underwent a cleaning process with magnetic beads and was denatured.

The raw data and FASTQ files, initially generated by the Illumina MiniSeq Sequencer (Illumina, San Diego, CA, USA), were transferred to a server for subsequent analysis. These files underwent processing with the MIA FORA FLEX version 5.2 alignment software. This software leveraged references from both the IPD-IMGT/Database version 3.50 and the Sirona Genomics database (Immucor, Norcross, GA, USA) to deduce HLA genotypes. The MIA FORA software version 5.2 executed a demultiplexing procedure based on unique indices and harnessed two complementary informatics algorithms: one for mapping sequences to references and another for de novo assembly. These algorithms collectively constructed one or two phased consensus sequences.

### 2.4. Statistical Analysis

In this study, we employed the MiDAS HLA [17] tool version 1.10.0, an R package integrated within the Bioconductor software version 3.18 suite, tailored for statistical association analysis. The MiDAS HLA package is equipped with immunogenetic data transformation functions, facilitating HLA amino acid fine mapping and the analysis of HLA evolutionary divergence. Our assessment of associations with HBV risk drew upon effect size estimates derived from a logistic regression model. These effect sizes, articulated as odds ratios, served to delineate the magnitude and directionality of the association. Both confidence intervals and measures of statistical significance were incorporated into our analysis. To adjust for multiple testing, we used the Benjamini and Hochberg (BH) correction method for *p*-values [18].

## 3. Results

The present study reports data from a single center experience in HLA typing using NGS in Romanian patients with chronic hepatitis B. Building on our previous experience, three-fields HLA was successfully achieved for all 11 loci of the HLA genes. The study included a total of 247 patients with chronic hepatitis B, consisting of 113 women aged 23 to 81 and 134 men aged 23 to 83. The control group featured 304 healthy individuals, with 118 women aged 19 to 48 and 186 men aged 18 to 50.

Table 1 summarizes HLA alleles’ impact on HBV risk through odds ratios derived from logistic regression. The odds ratios, representing both magnitude and direction of association, are accompanied by confidence intervals for statistical significance. Each allele is associated with a specific decrease in HBV risk, with all featured alleles indicating reduced risk. The “Estimate (95% CI)” column succinctly captures both effect size and accuracy, with odds ratios below one signifying a protective effect against HBV. In the chronic hepatitis B group, the HLA-DRB1*06:03:01 allele frequency was significantly lower compared to the normal control group, indicating a substantial correlation (OR = 0.19; 95% CI = 0.08–0.38, *p* = 0.002). This allele is present in about 4.90% of the total population, with a prevalence of 7.57% in the non-disease group and 1.62% in the disease group. Comparing allele distribution between the control and HBV groups, the frequency of the HLA-DRB1*13:01:01 allele was significantly higher in healthy subjects than in chronic HBV patients, suggesting a notable correlation (OR = 0.19; 95% CI = 0.08–0.41, *p* = 0.004). This allele was more prevalent in individuals without HBV (6.58%) compared to those in the HBV cohort (1.42%). Furthermore, the HLA-DQB1*06:02:01 allele occurred at a higher frequency in the non-disease group (6.09%) compared to the disease group (1.42%), implying a protective role against HBV infection (OR = 0.21; 95% CI = 0.08–0.45, *p* = 0.006). The HLA-DQA1*01:03:01 allele was less frequent in the HBV group than in the control group, with a significant correlation observed between the groups (OR = 0.38; 95% CI = 0.21–0.66, *p* = 0.018). This allele was more prevalent in non-HBV individuals, at 8.55%. The frequencies of HLA-DRB1*15:01:01 and HLA-DRB5*01:01:01 were notably higher in the non-disease group than in chronic HBV patients, indicating a protective effect against HBV infection.

To better understand the relationship between HLA genes at a two-field resolution and chronic hepatitis B infection, we utilized MiDAS-HLA for covariate adjustment, accounting for potential confounders. This approach provided a thorough and detailed analysis of their impact, as evidenced in Table 2.

The control group exhibited a higher frequency of the HLA-DQB1*06:03 allele compared to the HBV group, with a statistically significant difference (OR = 0.19; 95% CI = 0.08–0.38, *p* = 0.002). In our analysis, no other alleles were adjusted for in the model when assessing this particular allele. The HLA-DQB1*06:02 allele frequency was lower in CHB patients (1.42%) compared to the control group (6.09%). Notably, the DQB1*06:02 allele also demonstrated a protective association, with an odds ratio of 0.18. It is important to note that the effect size for this allele was adjusted to consider the presence of DQB1*06:03 as a covariate, indicating a possible interaction or linkage between these alleles. Despite this adjustment, DQB1*06:02 maintained its significance, with an adjusted *p*-value of 0.006.

Based on genotyping, we investigated the association-specific amino acid residues of each classical HLA gene with HBV susceptibility (Table 3). The associated amino acid positions were identified using conditional logistic regression analysis. HLA alleles at two-field resolution are defined by differences in their protein structure (one or many amino acids), resulting in very similar or very different antigen presentation profiles. Therefore, we analyzed amino acid positions in the peptide binding regions that have the same residue for one group of alleles but a different one for others.

The residue F at position DQB1_87 is notably associated with a significant decrease in HBV risk. With a *p*-value of <0.0001, the odds ratio of 0.175 (95% CI = 0.09–0.3042) signifies its protective nature against hepatitis B infection. Tracing this residue back to its originating HLA alleles, we find that it is exclusively linked to *06:02 and *06:03. Conversely, the residue Y at the same position, DQB1_87, leans towards an increased risk for hepatitis B, albeit with a *p*-value of 0.087, which is just outside the conventional threshold of significance. The odds ratio of 1.48 (95% CI = 1.06–2.08) hints at its potential role in elevating hepatitis B susceptibility. Mapping this residue to its source HLA alleles reveals a broader association, encompassing *05:01, *05:02, *05:04, *05:194, *06:04, and *06:09. The residue E, position DRB1_76, is associated with a marked decrease in hepatitis B risk, exhibiting an odds ratio of 0.27 (95% CI = 0.16–0.46) and an adjusted *p*-value <0.001. This residue is linked to HLA alleles *04:02, *11:02, *11:03, *13:01, and *13:02. Similarly, the residue A denotes a protective effect, with an odds ratio of 0.52 (95% CI = 0.32–0.84, *p* = 0.03). This residue is linked to HLA alleles *15:01 and *15:02. Amino acid variant I72 of HLA-DRB1 showcases a protective trend, with an odds ratio of 0.41 (95% CI = 0.29–0.58, *p* < 0.001). Conversely, the L residue, although presenting a higher odds ratio of 1.43 (95% CI = 1.01–2.03), lacks statistical significance, with a *p*-value of 0.1. Furthermore, DQB1_38 position for residue A is associated with a decreased hepatitis B risk, with an odds ratio of 0.40 (95% CI: 0.27, 0.58) and a significant adjusted *p*-value of *p* < 0.001. In contrast, the V residue leans towards an increased risk, with an odds ratio of 1.72 (95% CI = 1.14–2.62, *p* = 0.02). The L residue of DQB1_26 position showcases a protective association, with an odds ratio of 0.46 (95% CI = 0.32- 0.65, *p* < 0.001). Conversely, the G residue indicates an increased risk, with an odds ratio of 1.62 (95% CI = 1.15–2.29, *p* = 0.02). In addition, DQB1_9 position of the F residue stands out for its strong protective nature against CHB, as indicated by an odds ratio of 0.26 (95% CI = 0.12–0.49, *p* < 0.001). This residue is linked to HLA alleles *04:02 and *06:02.

## 4. Discussion

Multiple research investigations have demonstrated that distinct polymorphic variations in the human leukocyte antigen (HLA) class I and II gene clusters are integral to the immunological response, serving as pivotal genetic determinants for immune characteristics that govern the persistence or eradication of HBV. Notably, specific polymorphisms within HLA loci, with a particular emphasis on HLA-II, are correlated with spontaneous viral clearance or conferred immunity against HBV infection. In the context of the present study, an analysis identified that a subset of six HLA class II alleles exhibited a statistically significant correlation with enhanced protection against CHB infection.

Our research group previously showed that the most frequent HLA alleles found in the Romanian population are HLA-A*01, A*02, A*03, A*11, A*24; HLA-B*18, B*35, B*44, B*51; and HLA-DRB1*01, DRB1*03, DRB1*07, DRB1*11, DRB1*13, DRB1*15, DRB1*16 [19]. The group also found, in the same population, statistical correlations between certain pathologies, such as celiac disease and specific HLA haplotypes HLA-DQA1*05:01, HLA-DQB1*02:01, HLA-DQB1*02:02; DQA1*02:01-DQB1*02:02, DQA1*05:01-DQB1*02:01 [20].

The key findings of the present work are the identification of several HLA alleles associated with a significant protective effect against HBV, including DQB1*06:03:01, DRB1*13:01:01, DQB1*06:02:01, DQA1*01:03:01, DRB5*01:01:01, and DRB1*15:01:01. Additionally, these results are important when considered alongside previously published data involving different population groups and geographical areas.

In our population group, HLA-DRB1*13:01:01 exhibited the strongest association with a protective effect against CHB. Consistent with these findings, several studies previously reported negative associations between hepatitis B infection and HLA-DR13 [5,21,22]. Additionally, research by Ahn et al. suggests that HLA-DRB13 plays a role in the immune response and may contribute to the self-clearance of HBV [23]. The findings also correlate with other studies involving patients from Asia, USA, the Middle East, or Africa. For instance, DRB1*13:02 was linked to protection against persistent HBV infection in Gambian adults and children [15]. In Korea, an endemic area for HBV infection, various studies have reported associations between the HLA-DRB1*13 allele and protection against HBV as well as with viral clearance [24]. Following the same line, studies in a Turkish or Iranian populations showed a higher occurrence of HLA-DR13 in those developing spontaneous HBV antibodies, suggesting its protective effect against chronic HBV infection [11,25].

Another allele with potential protective roles against hepatitis B infection in Romanians is HLA-DRB1*15:01:01, a finding consistent with research done on other populations. A recent study on the Chinese population by Wang et al. indicated that HLA-DRB1*15:01 was more prevalent in the control group than in occult hepatitis B virus infection carriers [26]. Similarly, studies on the same population by Yang et al. identified HLA- DRB1*15 as the only allele linked to viral clearance [27]. Another study by Lu et al. found the DRB1*15/*16-DQA1*01:02 haplotype less common in chronic HBV patients compared to self-limited HBV infection groups [28]. Comparable results were observed in Iranian patients, where HLA-DRB1*15:01 was typically associated with protection against chronic hepatitis B infection, as reported by Baniaghil [25].

Our data also show that HLA-DQB1*06:02:01 and HLA-DQB1*06:03:01 alleles are more frequent in healthy groups when compared to chronic hepatitis B patients. Recent reports found in the literature for different world populations strengthen the validity of our findings. Thus, HLA-DQB1 alleles are associated with more favorable effects on the HBV vaccine [29], patients with a lower risk of developing chronic hepatitis B [30], protective roles in HBV infection in different populations and in the course of the disease [31,32,33,34], and positive immunological response to hepatitis B vaccination, correlating with higher antibody levels following HBV vaccine [35]. Naderi M et al. conducted a study on HLA-DQB1 polymorphisms across three generations (offspring, mother, and grandmother) of hepatitis B patients. They noted that HLA-DQB1*06:04 was exclusively present in the control group, implying its protective effect against HBV infection and its role in the spontaneous clearance of the virus [36]. Two meta-analysis studies also focus on the relationship between HLA-DQB1 and hepatitis B. They report that DRB1*13:01 and DQB1*06:02 expression can corelate with the antibody response to hepatitis B vaccine, showing high specificity but low sensitivity [37], and that DQB1*03 and HLA-DQB1*0602 have protective effects for hepatocellular carcinoma (HCC), whereas HLA-DQB1*02 and HLA-DQB1*05:02 have a higher risk for HCC occurrence [38].

The HLA-DQA1*01:03:01 allele was also significantly less expressed in our hepatitis B group versus healthy subjects. These findings differ from other studies done on populations from other geographical areas. For example, Xun et al. report that the frequency of the HLA-DQA1*01:02 allele in the chronic hepatitis B group was significantly lower than the frequency in the asymptomatic HBV carrier group [39]. Other studies are consistent with our data and point to a protective role of HLA-DQA1 in HBV infection: HLA-DQA1*0102 and HLA-DQA1*0104 were linked to a reduced risk of chronic HBV infection and the prevention of liver cirrhosis development in a Chinese population [40]; the frequency of the HLA-DQA1*01:02 allele was significantly lower in the chronic hepatitis B group compared to the group that experienced spontaneously resolved HBV infection [41]. Studies done on Indian or Taiwanese populations show that the DQA1*01:03 allele was notably more prevalent in individuals who spontaneously recovered from HBV compared to those with persistent infections, suggesting again a protective role of this allele against HBV infection [30,42]. In a Malaysian population, Riazalhossein et al. explored the potential association of HLA-DQA1 and HLA-DQB1 alleles and haplotypes with the progression of HBV infection to liver cirrhosis and hepatocellular carcinoma in the Malaysian population, but found no significant correlation [43]. 

Autoimmune hepatitis (AIH) is globally prevalent, affecting all ages, with varying rates in different regions and ethnic groups. Highly variable HLA genes are linked to diverse autoimmune diseases, including AIH, impacting the disease’s onset, clinical presentation, and treatment outcomes. The immune system component plays a fundamental role in AIH, with T cells subpopulations controlling the response to antigens having structural defects [44]. In Europe and North America, several haplotypes have been associated with the disease. Thus, in Northern Europe, AIH has been associated with DRB1*03 and DRB1*04 in adult patients [45]; Italian patients express HLA DR4 [46]; while in North America, DRB1*03:01-DQB1*02:01 and DRB1*04:01-DQB1*03:02 haplotypes were more present in AIH patients vs. healthy individuals [47].

Amino acid variations in class II HLA have been documented as significant factors in various infectious diseases, including CHB [48]. Several reports show that HLA-DRB1 leucine at position 26 displays a positive association with the HBV vaccine response, while arginine at position 4, aspartic acid at position 57, and tyrosine at position 60 and position 78 exhibit a strong protective effect against occult hepatitis B infection [26,49]. To the best of our knowledge, this is the first report on amino-acid residues in HLA class II and CHB in the Romanian population. All relevant findings are summarized in Table 3. Among other findings, we report that DQB1_87 amino acid residues have contrasting associations with hepatitis B risk—with phenylalanine appearing protective, while the presence of glycine suggests a potential risk increase. This mapping of HLA alleles provides insights for more focused genetic research and potential therapies. Recent global research progress on the roles of class I and II HLA molecules in HBV infection over the past 5 years are summarized in Table 4. 

One important limitation of the present work is the relatively limited number of participants involved. Another point of concern may be that this study did not report any data on acute hepatitis B patients. Future research should be conducted with a larger patient cohort and also with a focus on the acute stage of the disease.

## 5. Conclusions

In conclusion, our work identified for the first time, using high resolution methods, a range of HLA alleles that demonstrate a significant protective effect against HBV in Romanian patients. These included DQB1*06:03:01, DRB1*13:01:01, DQB1*06:02:01, DQA1*01:03:01, DRB5*01:01:01, and DRB1*15:01:01. The importance of these findings is highlighted when compared with previous research involving various demographic groups and regions globally. This comparison not only validates our results but also underscores the potential differences in genetic protection against HBV across diverse ethnicities and environments. Such insights are vital for developing more effective, region-specific strategies for HBV prevention and treatment.

## Figures and Tables

**Table 1:** Distribution of HLA gene polymorphisms in CHB group compared to control group. CHB—chronic hepatitis B; OR—odds ratio; CI—confidence interval; percentages express allele frequency; BH—Benjamini and Hochberg correction; *p* < 0.05—statistical significance; * *p* < 0.05; ** *p* < 0.01, as indicated *p* value after logistic regression.

HLA Allele	Estimate OR, (95% CI)	Adjusted *p*-Value (BH) with Significance	Total Percentage	Control Group	CHB Group
DQB1*06:03:01	0.19 (0.08, 0.38)	0.002 **	4.90%	7.57%	1.62%
DRB1*13:01:01	0.19 (0.08, 0.41)	0.004 **	4.26%	6.58%	1.42%
DQB1*06:02:01	0.21 (0.08, 0.45)	0.006 **	3.99%	6.09%	1.42%
DQA1*01:03:01	0.38 (0.21, 0.66)	0.018 *	6.35%	8.55%	3.64%
DRB5*01:01:01	0.38 (0.2, 0.67)	0.02 *	5.72%	7.73%	3.24%
DRB1*15:01:01	0.38 (0.2, 0.69)	0.02 *	5.63%	7.57%	3.24%

**Table 2:** HLA allele covariate results at two-field resolution with adjustments. CHB—chronic hepatitis B; OR—odds ratio; CI—confidence interval; percentages express allele frequency; BH—Benjamini and Hochberg correction; *p* < 0.05—statistical significance; ** *p* < 0.01, as indicated *p* value after logistic regression.

HLA Allele	Estimate OR, (95% CI)	Adjusted *p*-Value (BH) with Significance	Covariates Adjusted for	Total Percentage	Control Group	CHB Group
DQB1*06:03	0.19 (0.08, 0.38)	0.002 **		4.90%	7.57%	1.62%
DQB1*06:02	0.18 (0.07, 0.4)	0.006 **	DQB1*06:03	3.99%	6.09%	1.42%

**Table 3:** Associations of HLA class II amino acid residues with CHB. CHB—chronic hepatitis B; OR—odds ratio; CI—confidence interval; percentages express allele frequency; BH—Benjamini and Hochberg correction; *p* < 0.05—statistical significance; * *p* < 0.05; *** *p* < 0.001, as indicated *p* value after logistic regression; F—phenylalanine, Y—tyrosine, E—glutamic acid, A—alanine, I—isoleucine, L—leucine, V—valine, G—glycine.

Amino Acid Position	Amino Acid Residues	*p*-Value Adjusted *p*-Value (BH)	OR	95% CI	Explainable 2-Field Resolution Alleles	Frequency % CHB Group	Frequency % Control Group
DQB1_87	Y	0.087	1.48	1.06–2.08	*05:01, *05:02, *05:04, *05:194, *06:04, *06:09	134 (27.13%)	135 (22.20%)
DQB1_87	F	<0.0001 ***	0.175	0.09–0.3042	*06:02, *06:03	15 (3.04%)	82 (13.49%)
DRB1_76	E	<0.001 ***	0.27	0.16–0.46	*04:02, *11:02, *11:03, *13:01, *13:02	20 (4.05%)	74 (12.17%)
DRB1_76	A	0.03 *	0.52	0.32–0.84	*15:01, *15:02	28 (5.67%)	60 (9.87%)
DRB1_72	I	<0.001 *	0.41	0.29–0.58	*04:02, *07:01, *08:03, *11:02, *12:01, *13:01, *13:02, *13:03, *15:01, *15:02	92 (18.62%)	180 (29.61%)
DRB1_72	L	0.1	1.43	1.01–2.03	*01:01, *01:02, *03:01, *03:07, *04:01, *04:03, *04:04, *04:05, *04:07, *04:08, *10:01, *11:08, *11:42, *14:01, *14:04, *14:54, *16:02	163 (33.00%)	175 (28.78%)
DQB1_38	A	<0.001 ***	0.40	0.27–0.58	*03:01, *03:02, *03:03, *03:04, *03:05, *03:19, *04:02, *06:02, *06:03, *06:04, *06:06, *06:09	151 (30.57%)	243 (39.97%)
DQB1_38	V	0.02	1.72	1.14–2.62	*02:01, *02:02, *02:10, *05:01, *05:02, *05:03, *05:04, *05:194, *06:01	204 (41.30%)	223 (36.68%)
DQB1_26	L	<0.001 ***	0.46	0.32–0.65	*02:01, *02:02, *02:10, *03:02, *03:03, *06:02, *06:03, *06:04, *06:06, *06:09	126 (25.51%)	211 (34.7%)
DQB1_26	G	0.02	1.620	1.15–2.29	*03:05, *04:02, *05:01, *05:02, *05:03, *05:04, *05:194	155 (31.38%)	155 (25.49%)
DQB1_9	F	<0.001 ***	0.26	0.12–0.49	*04:02, *06:02	11 (2.23%)	47 (7.73%)

**Table 4:** New research on the roles of HLA class I and class II molecules in the occurrence and development of HBV infection across global populations within the past five years (SNP—single nucleotide polymorphism; SSP—sequence-specific primer; SSO—sequence-specific oligonucleotide; RT-PCR—Real-time PCR; SBT—sequence-based typing; NGS—next generation sequencing).

HLA Allele	Effect on HBV Infection	Study Population	Number (Case and Control)	Genotyping Method	References
A*30:01	Risk	Cameroonian	101	SBT	[50]
A*33:03	Risk	Japanese	989	Genome-wide SNP	[51]
A*34:02	Protective	Ghanaian	173	NGS	[52]
A*74:01	Protective	Ghanaian	173	NGS	[52]
B*13:02	Protective	Ghanaian	173	NGS	[52]
C*08:04	Protective	Ghanaian	173	NGS	[52]
C*03:04	Protective	Cameroonian	101	SBT	[50]
C*16:01	Risk	Ghanaian	173	NGS	[52]
DRB1*12	Protective	Burkinabè	144	Multiplex PCR	[53]
DRB1*11	Risk	Burkinabè	144	Multiplex PCR	[53]
DRB1*01	Protective	Transylvanian	160	RT-PCR	[16]
DRB1*03	Risk	Transylvanian	160	RT-PCR	[16]
DRB1*13:02	Risk	Taiwanese	15,352	Genome-wide SNP	[30]
DRB1*08:04	Protective	Ghanaian	173	NGS	[52]
DQB1*05	Risk	Transylvanian	160	RT-PCR	[16]
DQB1*06:09	Risk	Taiwanese	15,352	Genome-wide SNP	[30]
	Protective	Thai	647	Genome-wide SNP	[31]
DQB1*06:04	Protective	Iranian	148	SSP	[36]
DQB1*03:03	Risk	Iranian	148	SSP	[36]
DQB1*05:02	Risk	Iranian	148	SSP	[36]
DQB1*06:03	Protective	Chinese	689	SBT	[34]
DQB1*06:01	Risk	Japanese	1975	Genome-wide SNP	[54]
DPA1*01:03	Protective	Japanese, Korean, Chinese, Thai	3167	SSO	[6]
	Protective	Chinese	386	SBT	[55]
	Protective	Thai	647	Genome-wide SNP	[31]
DPA1*01:03:01	Protective	Taiwanese	840	SBT	[56]
DPA1*02:02	Risk	Japanese, Korean, Chinese, Thai	3167	SSO	[6]
	Risk	Thai	647	Genome-wide SNP	[31]
	Risk	Chinese	386	SBT	[55]
	Risk	Taiwanese	15,352	Genome-wide SNP	[30]
DPA1*02:02:02	Risk	Taiwanese	840	SBT	[56]
DPB1*02:01	Protective	Japanese, Korean, Chinese, Thai	3167	SSO	[6]
	Protective	Thai	647	Genome-wide SNP	[31]
	Protective	Japanese	989	Genome-wide SNP	[51]
DPB1*04:01	Protective	Japanese, Korean, Chinese, Thai	3167	SSO	[6]
DPB1*04:02	Protective	Japanese, Korean, Chinese, Thai	3167	SSO	[6]
	Protective	Japanese	1193	Genome-wide SNP	[57]
	Protective	Chinese	701	SBT	[58]
	Protective	Korean	283	SBT	[59]
DPB1*05:01	Risk	Japanese, Korean, Chinese, Thai	3167	SSO	[6]
	Risk	Thai	647	Genome-wide SNP	[31]
	Risk	Japanese	1193	Genome-wide SNP	[57]
	Risk	Chinese	701	SBT	[58]
	Risk	Korean	283	SBT	[59]
	Risk	Taiwanese	15,352	Genome-wide SNP	[30]
DPB1*09:01	Risk	Japanese, Korean, Chinese, Thai	3167	SSO	[6]
DQA1*01:02	Risk	Taiwanese	15,352	Genome-wide SNP	[30]

## Data Availability

Dataset available on request from the authors.
